# Global issues in aquaculture

**DOI:** 10.1093/af/vfae027

**Published:** 2024-09-05

**Authors:** Frank R Dunshea, Martin Sutcliffe, Hafiz A R Suleria, Shiba S Giri

**Affiliations:** School of Agriculture, Food and Ecosystem Sciences, Faculty of Science, The University of Melbourne, Parkville, Melbourne, VIC 3010, Australia; School of Biology, Faculty of Biological Sciences, The University of Leeds, Leeds LS2 9JT, UK; Centre for Innovation and Excellence in Livestock, Heslington, York YO10 5DG, UK; School of Agriculture, Food and Ecosystem Sciences, Faculty of Science, The University of Melbourne, Parkville, Melbourne, VIC 3010, Australia; ICAR-Central Institute of Freshwater Aquaculture, Bhubaneswar, India

Periodically, the World Association of Animal Production (WAAP) commissions a special Animal Frontiers issue on global matters; in this issue, the spotlight is on aquaculture. Aquaculture, or the farming of aquatic organisms such as fish, molluscs, crustaceans, and aquatic plants, is important both now and into the future for several key reasons. Aquaculture will be critical to meeting the global food demand as the human population is projected to reach 10 billion by 2050. This rising population in the face of limiting terrestrial resources will place increasing pressure on food production systems, and aquaculture offers a means of providing a significant source of protein-rich food, supplementing wild-caught fisheries, which are often overexploited. Fish and seafood are important sources of essential nutrients like omega-3 fatty acids, which benefit human health. Aquaculture can help meet the growing demand for these nutrients sustainably and, if practised responsibly, offers an opportunity to ensure food security and stability. Aquaculture can adapt to different environmental conditions and is less vulnerable to climate change than traditional agriculture. Species can be selected and managed to thrive in varying water temperatures and conditions. However, the aquaculture industry faces significant challenges and opportunities, some of which are highlighted in the following issue of *Animal Frontiers*. The need for further research and innovation in aquaculture practices is not just a suggestion but a necessity for the future of our food production systems.

Much of the increased population growth and demand will occur in Africa and South Asia. In the paper by Giri (2024), issues relating to aquaculture in South Asia, specifically India, Bangladesh, Nepal, Sri Lanka, Bhutan, Pakistan, Afghanistan, and Maldives, are not just reviewed but thoroughly examined. Their study provides a comprehensive understanding of farming practices in both freshwater and marine environments across specific regions. They review literature and empirical data to explore how farmers prepare and utilize homemade feeds. Factors such as the availability and cost-effectiveness of raw materials, feeding schedules, technological tools, and the challenges farmers face are analyzed. The research highlights the diverse reliance on locally sourced ingredients, influenced by each country’s socio-economic and environmental conditions. Additionally, the study investigated the adoption of commercial pellet feeds by small-scale farmers. These insights are not just significant but crucial for understanding sustainable aquaculture practices in South Asia, offering valuable implications for regional policy-making and research efforts aimed at enhancing food security and environmental sustainability. These data are not just informative but also have the potential to inform producers and policymakers in other parts of the world who are facing similar levels of expansion.

One area highlighted by Giri et al. (2024) was that despite the continued reliance on fish meal and oil, there was an ongoing exploration of alternative lipid and protein sources for aquafeeds as aquaculture competes with humans and other terrestrial livestock for these resources. In their paper, Selvaraj and Won (2024) discuss that insect farming has emerged as a promising avenue for sustainable aquaculture feed production, offering a reliable supply through controlled environment farming. However, the sector encounters substantial challenges such as scaling up production, automating processes, refining processing techniques, ensuring food safety and biosecurity, and navigating intricate market regulations. In many parts of the world, policymakers are also still having issues relating to the source material used to raise the insect and their larvae. This article delves into this growing industry, emphasizing the environmental benefits of insect-based feeds while highlighting the urgent requirement for targeted research, investment, and innovation to address the specific demands of aquafeed production. The challenges are not just significant, but urgent, and immediate action is needed to ensure the sustainability and growth of the aquaculture industry.

As the aquaculture industry moves toward some of these alternative protein sources, it is critical that the profile of these proteins can meet the specific needs of fish and other aquatic species. Suehs et al., (2024) highlight that glycine plays a crucial role as a functional amino acid in various physiological and metabolic processes essential for aquatic species. Recent studies have demonstrated additional benefits from supplemental dietary glycine in their diets. Future research should focus on understanding the biochemical mechanisms underlying glycine’s role in fish nutrition. Advances in aquaculture nutrition emphasize the importance of supplementing dietary glycine, traditionally considered “non-essential,” especially for fish consuming high-plant-protein diets. As aquaculture continues to expand globally, with a strategic shift from fishmeal to alternative ingredients, formerly classified “dispensable amino acids” like glycine are becoming key targets for further research. Optimizing glycine levels in aquatic diets, particularly for carnivorous species, is critical for enhancing the efficiency and profitability of modern aquaculture practices.

A consistent theme in this issue is the need for the correct policy setting for aquaculture to meet its unquestioned potential. Also, there is enormous jurisdictional variation in policy setting, which will most likely drive where aquaculture is practised, and this may not always be in the most favorable parts of the world. Focusing specifically on the United States, Hurley et al. (2024) highlight that regulatory inefficiencies and complex permitting processes pose significant barriers to marine aquaculture. Despite recent recommendations to ease these obstacles for prospective shellfish farmers, 2 critical issues remain unresolved. Firstly, many states have yet to adopt recommended improvements to their permitting frameworks, thereby perpetuating regulatory costs for shellfish growers. Secondly, the federal permitting system overseen by the U.S. Army Corps of Engineers faces legal challenges, leading to delays and uncertainty within the shellfish industry. As the United States seeks to expand its marine aquaculture sector, further research will be essential to streamline permitting systems, ensuring clarity and efficiency while maintaining rigorous environmental reviews.

## About the Authors

 Hafiz Suleria is an Associate Professor and ARC DECRA Fellow in the School of Agriculture, Food, and Ecosystem Sciences at the University of Melbourne. Before the ARC DECRA Fellowship, he had already completed his McKenzie Fellowship (UoM), Alfred Deakin Fellowship (Deakin University), and US Postdoctoral Fellowship (Kansas State University, USA) in the field of food science and nutrition. He completed his PhD at the University of Queensland (UQ) in collaboration with the Translational Research Institute (TRI), the UQ School of Medicine, and the Commonwealth Scientific and Industrial Research Organisation (CSIRO), Australia. Hafiz Suleria has a major research focus on food science and nutrition, particularly in screening phytochemicals and bioactive molecules from different plant, marine, and animal sources. His research interests include the isolation, purification, and characterization of bioactive compounds using various cutting-edge technologies, followed by their in vitro bioactivity, in vivo, cell culture, and animal studies. Hafiz Suleria has published more than 300 peer-reviewed scientific papers in different highly reputed and impacted journals. He is also in collaboration with more than five universities, working as a co-supervisor and special member for PhD students and involved in joint publications, projects, and grants.

 Dr. Shiba Shankar Giri leads the Fish Nutrition and Physiology Division at ICAR-Central Institute of Freshwater Aquaculture in Bhubaneswar, India. His research spans aquaculture nutrition, fish physiology, nutrigenomics, farm-made aquafeeds, and feeding standards for aquatic animals. With over 200 research papers, 20 book chapters, and a monograph to his credit, Dr. Giri has also edited ten aquaculture books for the South Asian Association for Regional Cooperation (SAARC). He spent three years as the Senior Program Specialist (Fisheries) at the SAARC Agriculture Centre in Dhaka, Bangladesh. Additionally, Dr Giri has served on the editorial boards for several journals, including Reviews in Aquaculture, Fishes, and the Indian Journal of Animal Nutrition. He also served as the Managing Editor of the SAARC Journal of Agriculture. He is a Fellow of the National Academy of Veterinary Science (India) and the Animal Nutrition Society of India.

 Martin Sutcliffe is the Aquaculture Innovation Lead at the UK Agri-Tech Centre, leading the organization's strategic and practical engagement with the aquaculture sector across the UK. As a trained PADI Dive Master and Reef Check Eco Diver, Martin has a deep passion for the ocean. He has a background in the public aquarium sector. He spent over 10 years working for Merlin Entertainments, designing innovative exhibits to engage visitors at SeaLife public aquaria around the globe with particular interest in live coral exhibits. This work has given him a deep understanding of how organisms interact with their environments, both in captivity and the wild. Martin has commercial aquaculture experience, working on Life Support System (LSS) design and hatchery development for Lumpfish. From 2017 to 2022, through the Dorset and East Devon Fisheries Local Action Group (FLAG) and the Dorset Aquaculture Development initiative, Martin worked to deliver community-led development projects and developed over 80 successful grant-funded projects across the aquaculture, fisheries, and seafood sectors. He chairs the South West Aquaculture Network (SWAN), the third pillar of Maritime UK South West’s work, aiming to develop cross-sectoral innovative projects in the wider maritime economy. Martin led the delivery of the Dorset Mariculture Strategy, which was developed in close consultation with Dorset's aquaculture sector. He provided sector expertise in support of the High Potential Opportunity from the Department for International Trade for Sustainable Aquaculture Development in Dorset. Martin led the development of the concept for an Aquaculture Innovation Hub for England, a national project defining which functions and services the Hub should provide.

 Frank Dunshea is a Redmond Barry Distinguished Professor and Chair of Agriculture at the University of Melbourne and Professor of Animal Growth and Development at the University of Leeds. He has a BAg Sci (Hons) and PhD from La Trobe University and postdoctoral training at Cornell University. He has more than 40 years of experience in growth physiology and nutrition and the use of domestic animals in nutritional and biomedical research. His research has had a high scientific impact, with many of the results being rapidly adopted by industry. Professor Dunshea is a respected research leader in global livestock industries and is committed to ensuring that livestock industries operate responsibly and sustainably. In addition to many awards, he is a Fellow of the Australian Nutrition Society, the Australasian Pig Science Association, the Australian Society of Animal Sciences, and the International Union of Nutritional Sciences. He is also President of the World Association for Animal Production. **Corresponding author**: fdunshea@unimelb.edu.au
